# Causal role of immune cells on risk of Parkinson’s disease: a Mendelian randomization study

**DOI:** 10.3389/fnagi.2024.1368374

**Published:** 2024-03-22

**Authors:** Jian Gu, Yue Qiao, Shuyan Cong

**Affiliations:** Department of Neurology, Shengjing Hospital of China Medical University, Shenyang, Liaoning, China

**Keywords:** Parkinson’s disease, immune cells, Mendelian randomization, neurodegenerative disease, causal inference

## Abstract

**Background:**

Previous observational studies have suggested a correlation between immune cells and Parkinson’s disease (PD), yet specific investigations into the causal relationship between the two remain limited. This study aims to explore this potential causal relationship.

**Methods:**

We utilized genome-wide association study (GWAS) data on immune cells and Parkinson’s Disease, conducting a two-sample Mendelian randomization (MR) analysis using single nucleotide polymorphisms (SNPs). To estimate causality, we employed inverse variance weighting (IVW), MR-Egger, and weighted median (WM) methods. For sensitivity analysis, we used Cochran’s Q-test, MR-Egger intercept, leave-one-out analysis, and funnel plots.

**Results:**

After false discovery rate (FDR) correction, the effects of PD on immune cells, and vice versa, were not statistically significant. These include CX3CR1 on CD14+ CD16-monocyte (OR = 0.91, 95% CI = 0.86–0.96, *p* = 0.0003 PFDR = 0.152), CD62L-CD86+ myeloid DC AC (OR = 0.93, 95% CI = 0.89–0.97, *p* = 0.0005, PFDR = 0.152),CD11b on Mo (OR = 1.08, 95% CI = 1.03–1.13, *p* = 0.001, PFDR = 0.152), CD38 on igd+ cd24− (OR = 1.14, 95% CI = 1.06–1.23, *p* = 0.001, PFDR = 0.152), D14+ cd16+ monocyte %monocyte (OR = 1.10, 95% CI = 1.04–1.17, *p* = 0.001, PFDR = 0.159). Additionally, PD may be causally related to the immune phenotype of CM CD8br %T cell (beta = 0.10, 95% CI = 1.14–1.16, *p* = 0.0004, PFDR = 0.151), SSC-A on monocyte (beta = 0.11, 95% CI = 1.15–1.18, *p* = 0.0004, PFDR = 0.1 SSC-A on monocyte). No pleiotropy was determined.

**Conclusion:**

This study suggested a potential causal link between immune cells and Parkinson’s Disease through the MR method, which could provide a new direction for the mechanistic research and clinical treatment of PD.

## 1 Introduction

Parkinson’s disease is a neurodegenerative disease of central nervous system (CNS) that can cause loss of dopamine-producing neurons in the substantia nigra (Braak and Del Tredici, 2008). The disease is characterized by four major motor symptoms: resting tremors, muscular rigidity, bradykinesia (slowness of movement), and postural instability (changes in posture) (Lee and Gilbert, 2016). In addition to the well-known motor symptoms, Parkinson’s disease can also lead to a range of non-motor symptoms such as Cognitive changes, Mood disorders, Sleep disturbances, Autonomic dysfunction and so on (Waskowiak et al., 2022). These symptoms result in the deterioration of the quality of life for patients (Wang et al., 2015). Furthermore, PD places a significant economic burden on not only the patients but also their families and society (Pan et al., 2005). Despite tremendous research efforts, no cure for PD has been discovered yet. As the global population ages, the incidence and prevalence of PD have been increasing year by year, posing a significant challenge to global health.

Recent scientific studies have stressed the essential role of the immune system in the development of Parkinson’s disease (PD) (Galiano-Landeira et al., 2020; Tan et al., 2020; Bloem et al., 2021). A strong association between PD and circulating immune cells is reported, specifically lymphocytes and monocytes (Morato Torres et al., 2020). These cells, which roam the body, react to inflammation and damage in various areas. They possess the ability to breach the blood–brain barrier, penetrate the brain, and potentially contribute to its functioning. Some PD patients have been observed that certain immune cell activities increasing may damage dopaminergic neurons (Barcia, 2013). For example, T cells may take part in targeting neurons, thereby accelerating disease progression (Garretti et al., 2022). Additionally, the presence of aberrant levels of specific immune markers in the bloodstream of individuals with Parkinson’s disease suggests a close association between immune system dysregulation and the progression of the disease. Previous studies have found that people with PD have lower lymphocyte counts than controls due to reduced counts of helper cd4+ T cells and B cells (Gruden et al., 2011; Stevens et al., 2012). Some case–control studies also found that patients with confirmed PD had higher neutrophil and lymphocyte counts compared to controls (Akıl et al., 2015). Evidence from genetic, epidemiological, and cytokine profile studies further supports the notion that immune dysregulation may contribute to the pathogenesis of PD (Tan et al., 2020). It is critical to acknowledge that much of the existing evidence on the relationship between circulating immune cell counts and the onset of PD is derived from observational studies. These studies, while informative, may be subject to confounding factors and reverse causality. Consequently, the causal linkage between these immunological markers and Parkinson’s disease onset remains under-explored. Further research utilizing more robust, ideally experimental, methodologies is necessary to establish a definitive causal relationship.

Mendelian randomization (MR) emerged as an alternative to randomized controlled trials (RCTs) to establish reliable causal evidence between exposure and outcome by utilizing genetic variation (Smith and Ebrahim, 2003). This approach is effective in determining causality independently of confounders and avoiding reverse causality, as gene variants are randomly assigned at conception prior to the onset of disease (Davey Smith and Hemani, 2014). Previous studies using two-sample MR had shown a causal link between BMI /serum iron levels and the risk of PD (Pichler et al., 2013; Noyce et al., 2017). Previous observational studies have identified a range of associations between characteristics of immune cells and Parkinson’s disease (PD), lending support to the hypothesis of a link between these two factors. To explore this potential connection further, the present study employs a comprehensive two-sample MR analysis. This approach is designed to determine the causal relationship, if any, between the characteristics of immune cells and the development of PD, thereby advancing our understanding of the immunological underpinnings of this neurodegenerative disease.

## 2 Materials and methods

### 2.1 Study design

**Figure 1:**
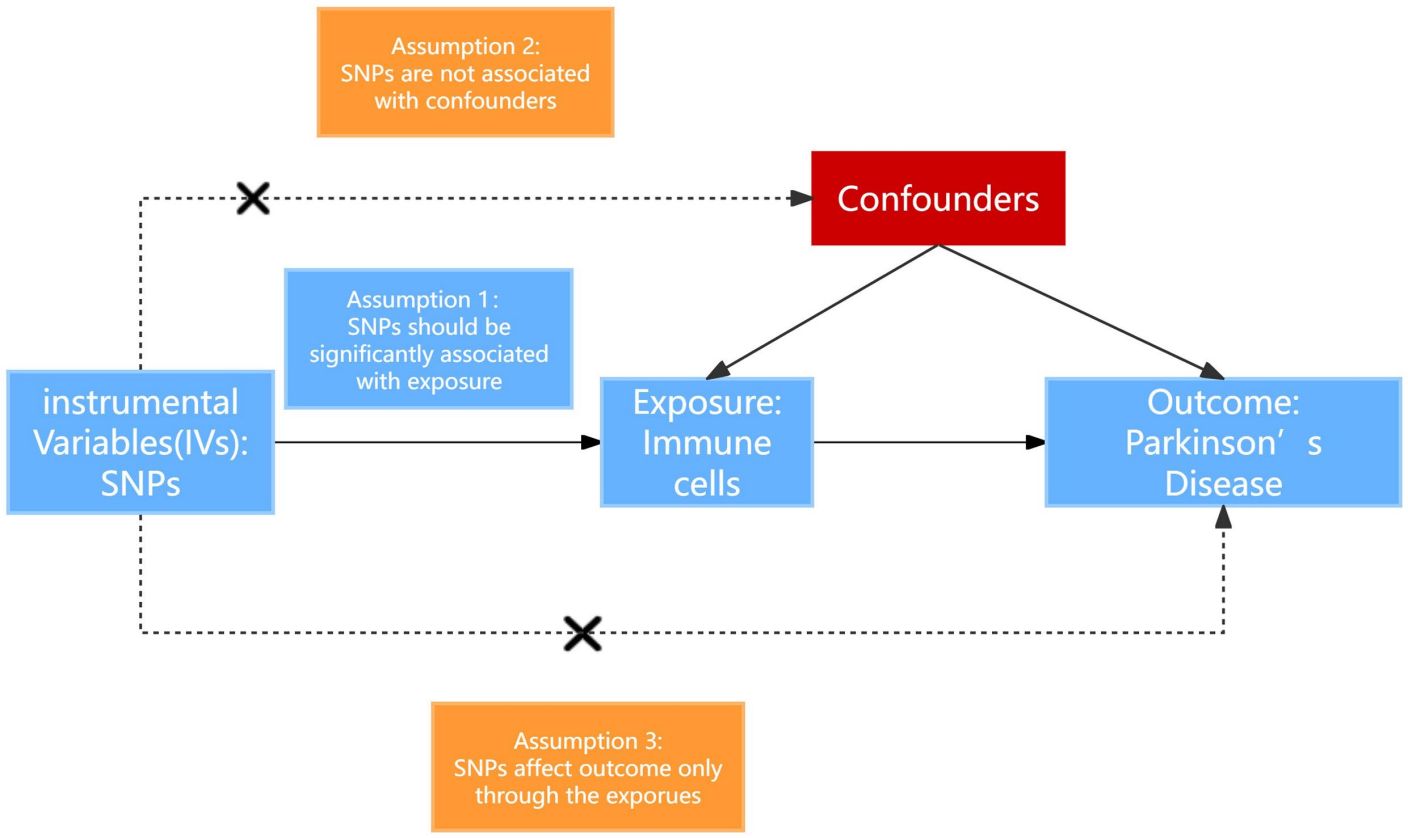
Schematic diagram of MR analysis. SNP, single nucleotide polymorphisms.

In our investigation, we implemented a two-sample MR analysis to assess the causal relationship between a set of 731 immune cell signatures which categorized into seven distinct groups and the PD. This methodology utilizes genetic variations as proxies for risk factors. For the validity of our chosen instrumental variables in this analysis, they must satisfy three fundamental criteria: (1) The genetic variants selected must exhibit a direct and significant association with the exposure; (2) these genetic variants must not be associated with potential confounders that could obscure or confound the relationship between the exposure and the outcome (Parkinson’s disease); (3) the influence of the genetic variants on the outcome should occur solely through the exposure, without involving alternative pathways (Gagliano Taliun and Evans, 2021). The overall design is shown in Figure 1.

### 2.2 Immunity-wide GWAS data sources

For exposure instrument, we publicly obtained summary statistics of blood cell traits from the GWAS catalog (accession numbers gcst90001391 to gcst90002121) (Orrù et al., 2020). The data included 563,085 participants of European ancestry and 731 immunophenotypes were included. Genetic variants linked to the levels of circulating leukocytes, lymphocytes, monocytes, neutrophils, eosinophils, and basophils were identified and utilized in this study. The data encompassed a range of immune cell characteristics, including absolute cell count (AC), median fluorescence intensity (MFI) which reflects the level of surface antigens, morphological parameters (MP), and relative cell count (RC). Specifically, the MFI, AC, and RC data encompassed various immune cell types such as B cells, CDCs (dendritic cells), mature T cells, monocytes, bone marrow cells, TBNK cells (T cells, B cells, natural killer cells), and Treg (regulatory T) cells. Meanwhile, the MP data included information on CDCs and TBNK cells. The reference panel based on the latest aggregated GWAS data including 3,757 Sardinians was used (Sidore et al., 2015).

### 2.3 Genome-wide association study data sources for PD

GWAS summary statistics for PD were obtained from the International Parkinson’s Disease Genomics Consortium (Nalls et al., 2019). The consortium executed a genome-wide association study (GWAS) on a European cohort of 482,730 individuals, consisting of 33,674 PD cases and 449,056 controls. Post stringent quality control and imputation, the study analyzed approximately 17.9 million genetic variants. This GWAS identified 90 independent genome-wide significant signals across 78 loci, including 38 independent risk signals in 37 novel loci. These variants explained 26–36% of the heritable risk of PD (Nalls et al., 2019).

### 2.4 Selection of genetic instruments

We selected SNPs associated with the exposure under genome-wide significance threshold and 1 × 10^−5^ was set to be the significance level of instrumental variables (IVs) for each immune trace. We employed the clumping process using PLINK software (version v1.90) to selectively prune the identified SNPs. This was carried out under stringent criteria, setting the linkage disequilibrium (LD) r2 threshold to less than 0.001 within a 1,000 kb distance (Auton et al., 2015). We calculated the F-statistic for each IV to avoid weak instrumental bias and evaluate the strength of its association with the exposure (Pierce et al., 2011), then removed IVs with an F statistic <10 (Brion et al., 2013). To ensure that the observed association effects corresponded to the same alleles, we excluded SNPs from our analysis.

### 2.5 Statistical analysis

In our analysis, we used five effective methods [MR-Egger; Bowden et al., 2015, weighted median Bowden et al., 2016, inverse variance weighted (IVW) Burgess et al., 2017, simple mode, and weighted mode] to deduce the potential causal relationships between PD and 731 immunophenotypes. The IVW method yielded the most reliable outcomes in the absence of horizontal pleiotropy among the IVs (Huang et al., 2021). We used the IVW which evaluates the causal influence of genetically predicted exposures on outcomes by weighted regression of SNP-specific Wald ratios as the main approach. Heterogeneity *p*-values are based on the Cochran’s Q statistic, and *p* < 0.05 indicates that single nucleotide polymorphisms were considered to exist heterogeneity. Consistent estimations of the causal effect can be achieved through the weighted median method when a minimum of half of the SNPs serve as effective instrumental variables (Bowden et al., 2016). The directional horizontal pleiotropy effect can be evaluated by the MR-Egger method through the intercept term that if the intercept term has a statistical difference with zero, it means that horizontal pleiotropy is affecting the results of an MR analysis (Burgess and Thompson, 2017). If the effect directions derived from the MR-Egger and IVW methods are consistent, it will increase the credibility of the results (Slob and Burgess, 2020). The simple mode does not weigh the genetic variants based on their precision or effect on the exposure, potentially leading to less stable estimates when individual variants have varying directions or magnitudes of effect (Sun et al., 2021). Simple mode and weighted mode were conducted as complementary analytical approaches. The MR pleiotropy residuals sum and outliers (MR-PRESSO) method was used to detect and remove outliers that severely affect the estimation results to reduce bias (Verbanck et al., 2018). After conducting MR analysis using the IVW method for the 731 types of immune cells included as exposure factors, we applied Benjamini-Hochberg (B-H) correction to the *p*-values obtained. We set the FDR correction threshold at 0.2 as the criterion to determine the presence of a causal relationship between the exposure and the outcome variable. We used leave-one-out analysis to explore the stability of these genetic variants by removing one of the selected individual SNPs each time (Wu et al., 2020). We also used the Scatter plots to ensured that these results were not driven by outliers. The robustness of the correlation and no heterogeneity were proved through Funnel plots. All statistical and sensitivity analyses were carried out using R statistical software (version 4.3.1) with the TwoSample MR package, MR (0.5.6), and MR PRESSO packages.

## 3 Results

### 3.1 The causal effect and sensitivity analysis of immunophenotypes on PD

**Figure 2:**
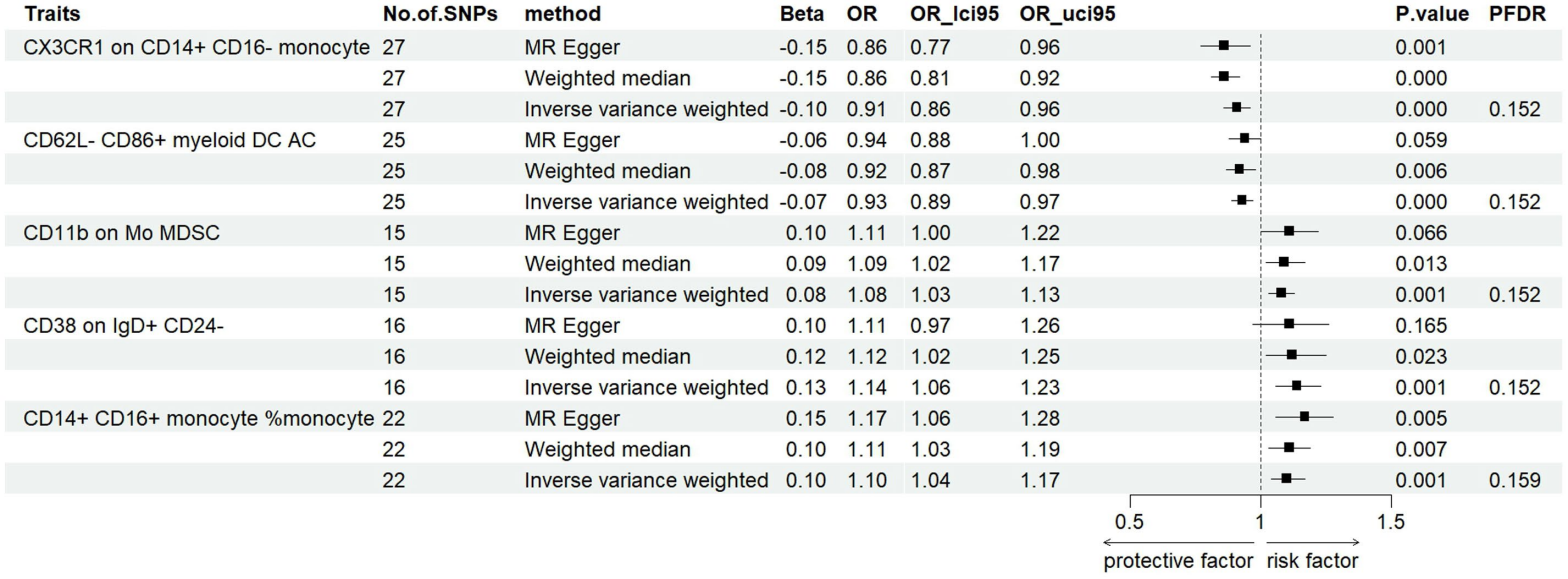
Forest plots showed the causal associations between PD and immune cell traits (FDR < 0.2).

To assess the effect of immunophenotype on PD risk, we used IVW as the main analysis method to perform MR analysis of two samples. We used the Benjamini-Hochberg FDR method to correct the *p*-value of multiple tests. After multiple test adjustment, no immune trait was identified when the significance threshold was set at *p* < 0.05 (Keselman et al., 2002). We identified three suggestive immunophenotypes associated with PD that were risk factors for PD (FDR < 0.2): CD11b on Mo MDSC (myeloid cell panel), CD38 on IgD+ CD24− (B cell panel) and CD14+ CD16+ monocyte %monocyte (monocyte panel) (Supplementary Figures S1–S4). We identified CX3CR1 on CD14 + CD16-monocyte (monocyte panel) and CD62L-CD86+ myeloid DC AC (cDC panel) as protective factors for PD (FDR < 0.2) (Supplementary Figures S1–S4). Our study’s findings indicate a significant causal association between the risk of Parkinson’s disease (PD) and an elevated level of CD11b on monocytes (Mo). This was evidenced by the inverse variance weighted (IVW) analysis (OR = 1.08, 95% CI = 1.03–1.13, *p* = 0.001, PFDR = 0.152). This association was further supported by similar outcomes from MR-Egger (OR = 1.11, 95% CI = 1.00–1.22, *p* = 0.066) and weighted median (WM) analyses (OR = 1.09, 95% CI = 1.02–1.17, *p* = 0.013) (Figure 2). Additionally, we investigated the impact of CD38 on IgD+ CD24-on PD risk using the IVW method, which suggested a significant correlation (OR = 1.14, 95% CI = 1.06–1.23, *p* = 0.001, PFDR = 0.152). Complementary results from MR-Egger (OR = 1.11, 95% CI = 0.97–1.26, *p* = 0.165) and WM analyses (OR = 1.12, 95% CI = 1.02–1.25, *p* = 0.023) reinforced this finding (Figure 2). Further analysis of CD14+ CD16+ monocyte percentage in relation to PD risk, again using the IVW method, indicated a notable association (OR = 1.10, 95% CI = 1.04–1.17, *p* = 0.001, PFDR = 0.159). MR-Egger (OR = 1.17, 95% CI = 1.06–1.28, *p* = 0.005) and WM analyses (OR = 1.11, 95% CI = 1.03–1.19, *p* = 0.007) corroborated these results, respectively, both showing statistical significance (Figure 2). In the case of CX3CR1 on CD14+ CD16-monocyte with respect to PD risk, the IVW method revealed a significant relationship (OR = 0.91, 95% CI = 0.86–0.96, *p* = 0.0003 PFDR = 0.152). This finding was supported by MR-Egger (OR = 0.86, 95% CI = 0.77–0.96, *p* = 0.001), WM analysis indicated a stronger correlation (OR = 0.86, 95% CI = 0.81–0.92, *p* = 0.02) (Figure 2). Furthermore, our analysis of CD62L-CD86+ myeloid DC AC in PD risk, as assessed by the IVW method, showed a negative association (OR = 0.93, 95% CI = 0.89–0.97, *p* = 0.0005, PFDR = 0.152) and WM analyses showed similar result (OR = 0.92, 95% CI = 0.8–0.98, *p* = 0.006). While MR-Egger analysis showed a non-significant trend (OR = 0.94, 95% CI = 0.88–1, *p* = 0.059) (Figure 2). Robustness tests including Cochran’s Q test, MR Egger regression, MR-PRESSO, and the leave-one-out test were conducted. These tests confirmed that the MR estimates were relatively robust, lending further credibility to our findings. Detailed information is shown in Supplementary Tables S1–S4.

### 3.2 The causal effect and sensitivity analysis of PD onset on immunophenotypes

**Figure 3:**
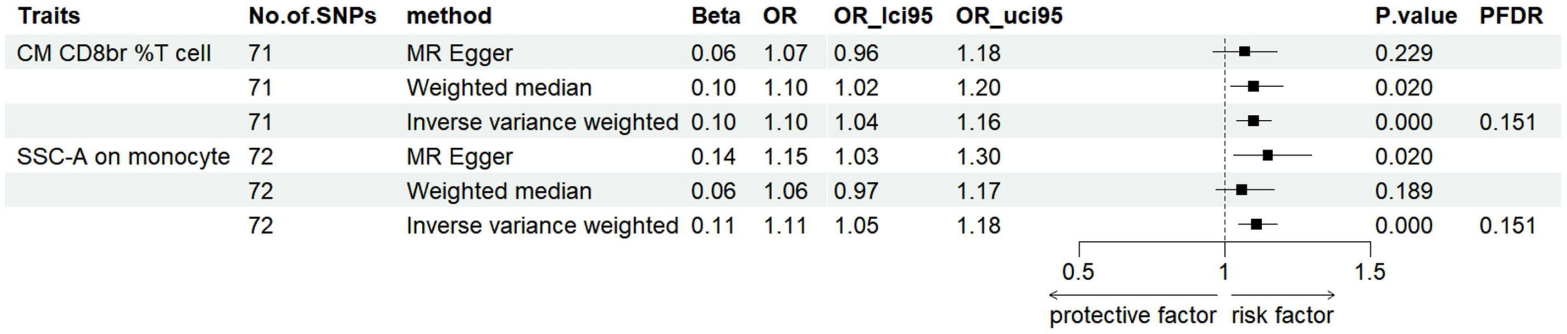
Forest plots showed the causal associations between immune cell traits and PD by using different methods (FDR < 0.2).

To investigate the causal influence of PD onset on immune cell phenotypes, we applied the same analytical methods used to evaluate the causal effect of immunophenotypes on PD. After the selection and harmonization of IVs, we utilized 71 SNPs for CM CD8br %T cell and 72 SNPs for SSC-A on monocytes for MR analysis. Our data revealed a significant increase in CM CD8br %T cell levels post-PD onset (FDR < 0.2) (Supplementary Figures S5–S8), with a beta coefficient of 0.10 and 95% CI of 1.04–1.16 (*p* = 0.0004, PFDR = 0.151). This finding was supported by WM analysis, which showed beta values of 0.10 (95% CI = 1.02–1.20, *p* = 0.02). While MR-Egger analysis showed a non-significant trend (beta = 0.06, 95% CI = 0.95–1.18, *p* = 0.229) (Figure 3). Moreover, an increase in SSC-A levels on monocytes was observed in PD patients, indicated by a beta coefficient of 0.11 (95% CI = 1.05–1.18, *p* = 0.0004, PFDR = 0.151). This trend was consistent with results from MR-Egger method (beta = 0.14, 95% CI = 1.03–1.30, *p* = 0.02) analyses. The results obtained by WM method were not statistically significant (beta = 0.06, 95% CI =0.97–1.17, *p* = 0.18) (Figure 3; Supplementary Tables S3, S4). These findings collectively suggest a potential causal relationship between PD onset and alterations in specific immunophenotypes. Detailed information is shown in Supplementary Tables S5–S7.

## 4 Discussion

In this study, we evaluated the causal relationship between immune cells and PD by two-sample bidirectional MR method after consulting the newly published literature of 731 immune cell genetic data. To date, there has been one published article on MR concerning neurodegenerative diseases and immune cells (Tang et al., 2024) however, the selection of instrumental variables and correction methods performed in our study were different, aiming to conduct a more comprehensive investigation into the possible causal relationship between immune cells and PD. Furthermore, we analyzed the potential reverse causality of 731 immune cells with PD, allowing for a more comprehensive analysis of subtle reverse trends to provide different perspectives. According to our study, we found that five immune phenotypes have causal effects on PD, of which three are risk factors for PD and two are protective factors. In addition, we found that PD has a causal protective effect on the 2 phenotypes (FDR < 0.20).

Our study found that the risk of PD increased with the increase of CD11b on Mo MDSC. myeloid derived suppressor cells (MDSC) are immunosuppressive cells that play a key role in a variety of biological processes. CD11b is a cell surface protein that belongs to the integrin family and is usually expressed on the surface of leukocytes (such as monocytes). It plays a role in cell adhesion, migration and phagocytosis. MDSCs are divided into two main types, namely neutrophil like MDSC (PMN-MDSC) and monocyte like MDSC (Mo MDSC). MDSC is similar to monocytes, expressing CD11b and other markers of monocytes. Yang et al. (2017) and Chen et al. (2017) analyzed MDSCs from 80 and 18 PD patients, respectively. Compared with the control group, the number of MDSCs in the peripheral circulation of PD patients was significantly increased. This suggests that MDSCs may be an important factor in the occurrence and development of PD. MDSCs have the potential to serve as effective biomarkers for PD diagnosis. Gopinath et al. (2022) performed an immunophenotypic analysis of blood samples from PD patients and observed the presence of myeloid-derived suppressor cells (MDSCs), with a significant increase in monocyte MDSCs while granulocyte MDSCs remained unchanged. Previous studies have shown that Mo MDSC has a certain positive correlation with PD. We further speculate that Mo MDSC has a causal relationship with PD through MR method, and we can further study its specific mechanism. Interleukin-10 (IL-10) has been implicated in the suppression of pathogenic inflammation and promotion of peripheral tolerance to inflammation (Yu et al., 2017). Meanwhile, regulatory B cells (Bregs), which are highly enriched in CD24+ CD38+ cells, express IL-10 (Zeng et al., 2022). We conclude that negative expression of CD24 may lead to insufficient secretion of IL-10, which may lead to decreased anti-inflammatory ability of cells and further induce PD, but more experiments are needed to verify this. Specific studies directly correlating CD14+ CD16+ monocytes with PD were not found in the search, it is known that immune system alterations, including changes in monocyte populations, are associated with PD. It has been found that leucine rich repeat kinase 2 (LRRK2) is associated with PD. In some populations, more than 30% of PD patients carry the G2019S mutation (Cookson, 2010). Thévenet et al. found that the expression level of LRRK2 protein in CD14+ CD16+ cells was higher than that in CD14+ CD16-cells by analyzing the expression of LRRK2 mRNA and protein in human peripheral blood mononuclear cells (PBMCs) subpopulation (Thévenet et al., 2011). We speculate that CD14+ CD16+ monocyte% monocyte affects the pathogenesis of PD by increasing the expression of LRRK2, and the specific mechanism needs further experimental support.

We found that CD62L-CD86+ myeloid DC AC and CX3CR1 on CD14+ CD16-monocyte are protective factors for PD. CD62L-CD86+ myeloid dendritic cells are important immune modulatory cells that can affect the activation and suppression of the immune system. In Parkinson’s Disease, abnormal activation of the immune system is considered one of the key factors in disease progression. Through the action of their surface molecule CD86, these dendritic cells can promote the activation and differentiation of T cells, thereby affecting the immune response in Parkinson’s Disease. Moreover, some studies suggest that immune cells, including dendritic cells, can release neuroprotective factors involved in protecting neuronal cells from damage (Wang et al., 2024). Therefore, the absolute count of CD62L-CD86+ myeloid dendritic cells may reflect the state of immune dysregulation in Parkinson’s Disease. CX3CR1 is a receptor expressed on various immune cells, including monocytes, microglia, macrophages, dendritic cells, T cells, and natural killer (NK) cells. In the context of Parkinson’s disease (PD), the CX3CR1 receptor and its ligand, the chemokine fractalkine (CX3CL1), are of significant interest. The CX3CL1-CX3CR1 interaction plays a pivotal role in modulating microglial activity, a crucial element in the neuroinflammatory processes associated with PD. Castro-Sánchez et al. (2018) suggest that CX3CR1 signaling may exert a protective effect against neuroinflammation and neurodegeneration in PD, particularly in relation to alpha-synuclein pathology, a characteristic feature of the disease. Further research is essential to unravel the intricacies of CX3CR1 signaling in PD, potentially leading to novel strategies for treatment or management of the disease.

In addition, our study indicated that the onset of PD might be causally related to the increase of CM CD8br %T cell and SSC-A on monocyte levels. Research indicates that T cells, including CD8+ T lymphocytes, play a key role in the pathogenesis of PD (Baird et al., 2019). T cell dysregulation is closely related to disease progression and severity. The relationship between CM CD8br %T cells and Parkinson’s disease (PD) needs to be further explored in the future. SSC-A on monocytes refers to the measurement of side scatter area during flow cytometry and it is useful for identifying monocyte subpopulations and understanding their role in immune responses and disease. Changes in monocyte activation and function can be detected by parameters such as SSC-A, and the progression of neurodegenerative diseases may be evaluated by changes in SSC-A due to the interaction of the immune system with the CNS.

However, several limitations are worth mentioning. First, this study is based on genome-wide association data from Europe, so it is necessary to be cautious about whether the interpretation and analysis of the conclusions can be extended to other ethnic groups. Secondly, it is important to acknowledge the inherent limitations of MR analysis due to epigenetic factors. These include issues such as DNA methylation, RNA editing, and the activity of transposable elements, all of which can influence gene expression and function independently of the genetic variants used as instrumental variables in MR studies. Thirdly, despite utilizing the most comprehensive and up-to-date GWAS database available, the sample size of our study remains relatively small when compared to large-scale, population-based observational studies. This limitation may affect the robustness and generalizability of our findings, and it underscores the need for further research with larger sample sizes to validate and extend our results. Fourth, we adjusted the threshold setting used to evaluate the results to *p* < 0.2, which may lead to some false positives, but it can more comprehensively include immune cell types that may be causally related to PD.

## 5 Conclusion

In conclusion, we performed a two sample MR analysis of the results of a recently published large GWAS cohort using genetic instrumental variables. Our results showed that PD was causally related to several immune phenotypes, excluding the influence of confounding factors and reverse causality. This may help to explore the physiological and pathological mechanisms of PD, and provide new clues for early prevention and intervention of PD.

## Data availability statement

The original contributions presented in the study are included in the article/Supplementary material, further inquiries can be directed to the corresponding author.

## Author contributions

JG: Conceptualization, Data curation, Formal analysis, Investigation, Methodology, Software, Writing – original draft, Writing – review & editing. YQ: Formal analysis, Methodology, Writing – review & editing. SC: Conceptualization, Funding acquisition, Supervision, Writing – review & editing.
