# Supplementary material for: Causal role of immune cells on risk of Parkinson’s disease: a Mendelian randomization study
Source: Front Aging Neurosci. 2024 Mar 22;16:1368374. doi: 10.3389/fnagi.2024.1368374 (PMC10995297; doi:10.3389/fnagi.2024.1368374)

Figure S1. Scatter plot of immunophenotypes on PD. (A) CX3CR1 on CD14+CD16- monocyte, (B) CD62L-CD86+ myeloid DC AC, (C) CD11b on Mo MDSC , (D) CD38 on IgD+ CD24-, (E) CD14+ CD16+ monocyte %monocyte. Three lines reveal the estimated effect sizes by MR methods (inverse-variance weighted, MR-Egger and weighted median) .

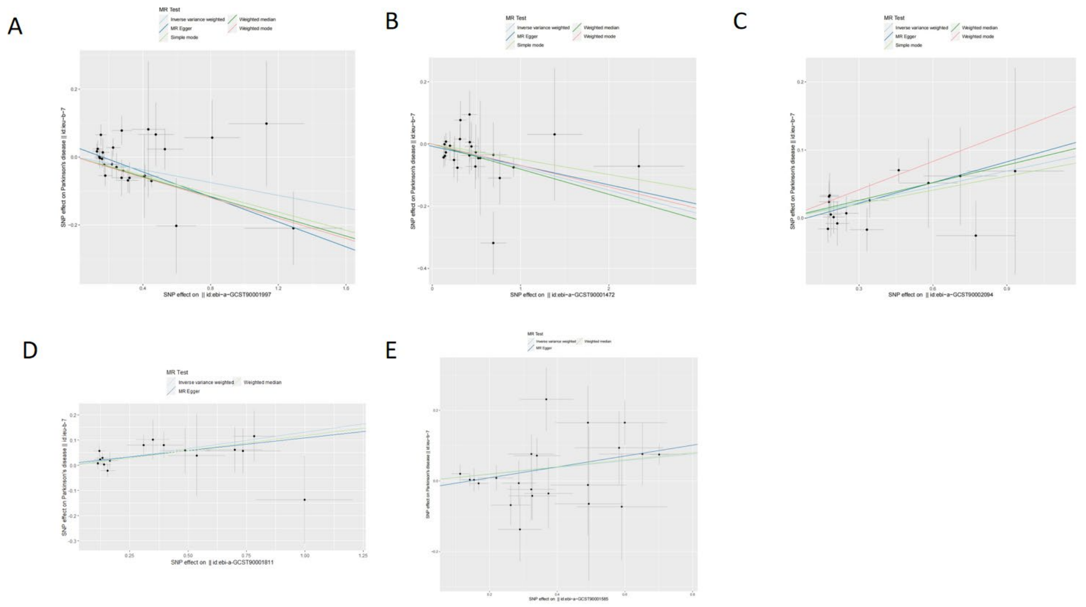

Figure S2. Funnel plot of immunophenotypes on PD. (A) CX3CR1 on CD14+CD16- monocyte, (B) CD62L-CD86+ myeloid DC AC, (C) CD11b on Mo MDSC , (D) CD38 on IgD+ CD24-, (E) CD14+ CD16+ monocyte %monocyte.

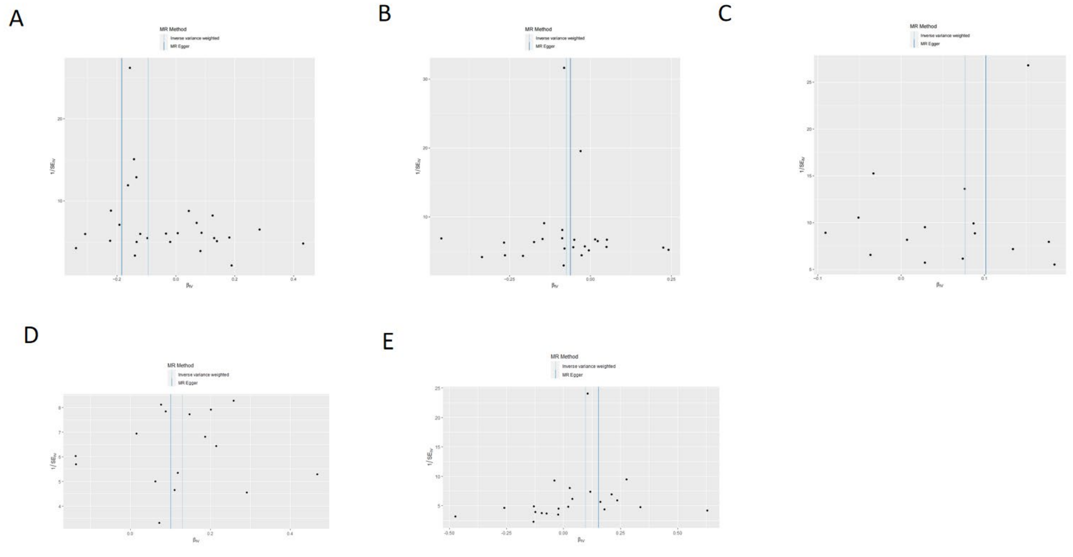

Figure S3. Forest plot of immunophenotypes on PD. (A) CX3CR1 on CD14+CD16- monocyte, (B) CD62L-CD86+ myeloid DC AC, (C) CD11b on Mo MDSC , (D) CD38 on IgD+ CD24-, (E) CD14+ CD16+ monocyte %monocyte.

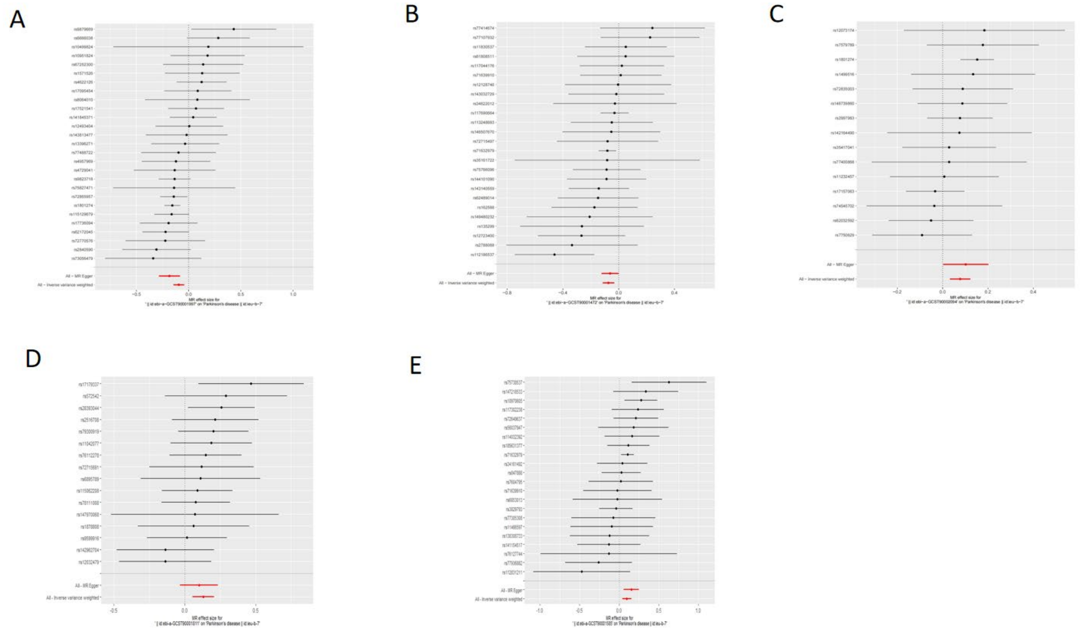

Figure S4. Leave-one-out analysis result of immunophenotypes on PD. (A) CX3CR1 on CD14+CD16-monocyte, (B) CD62L-CD86+ myeloid DC AC, (C) CD11b on Mo MDSC , (D) CD38 on IgD+ CD24-, (E) CD14+ CD16+ monocyte %monocyte.

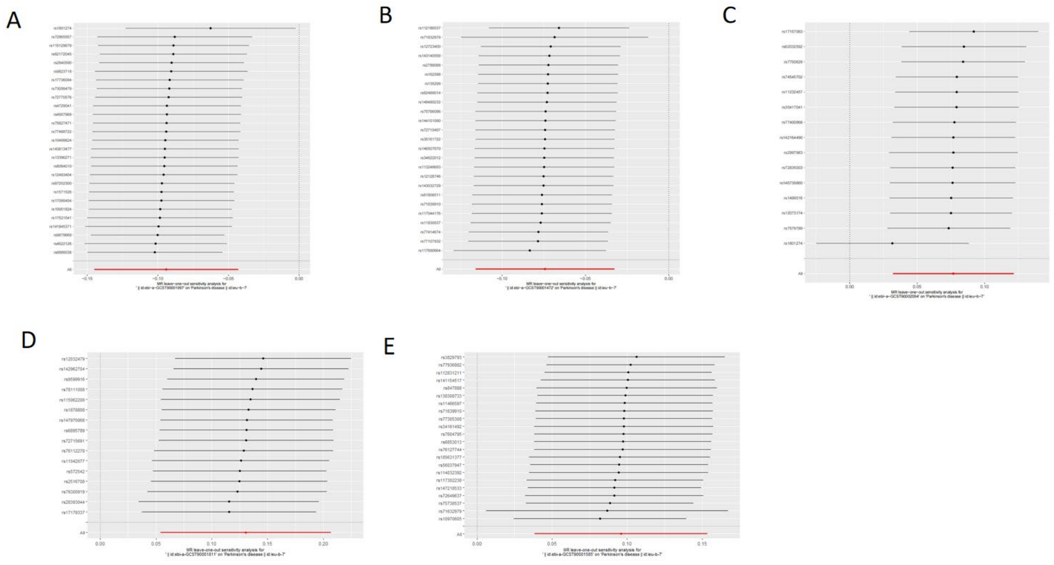

Figure S5. Scatter plot of PD on immunophenotypes. (A) CM CD8br %T cell , (B) SSC-A on monocyte.

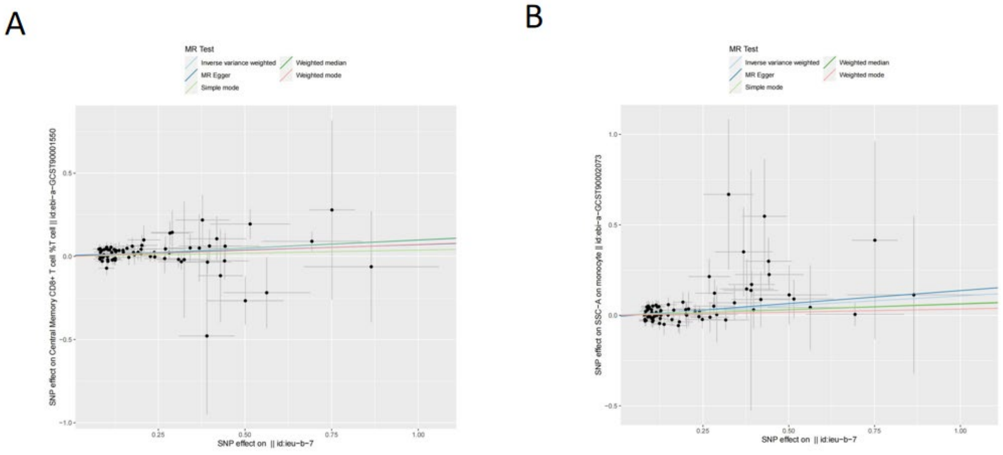

Figure S6. Funnel plot of PD on immunophenotypes. (A) CM CD8br %T cell , (B) SSC-A on monocyte.

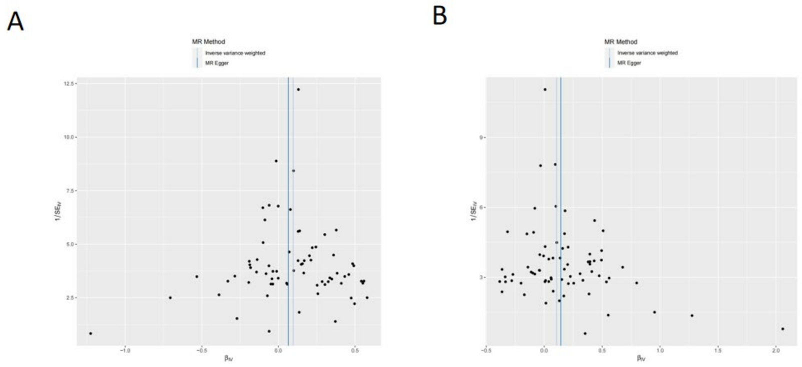

Figure S7. Forest plot of PD on immunophenotypes. (A) CM CD8br %T cell , (B) SSC-A on monocyte.

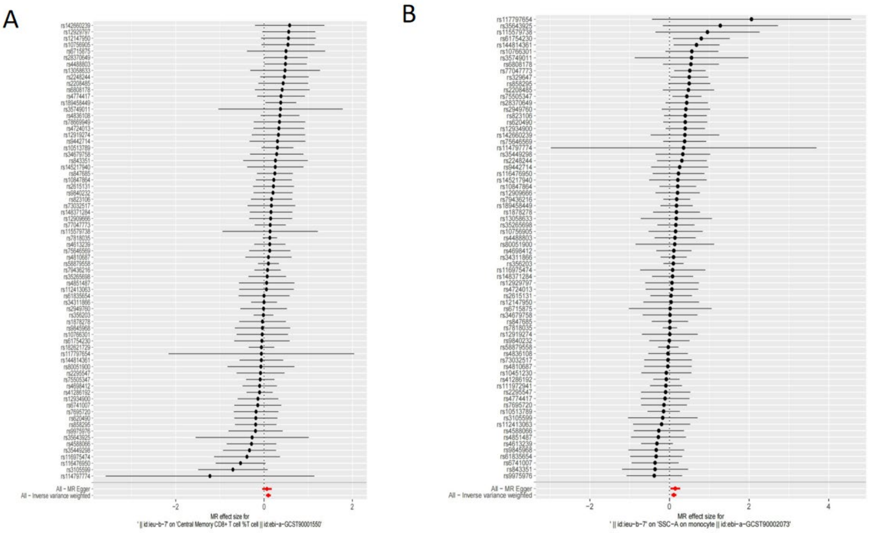

Figure S8. Leave-one-out analysis result of PD on immunophenotypes. (A) CM CD8br %T cell , (B) SSC-A on monocyte.

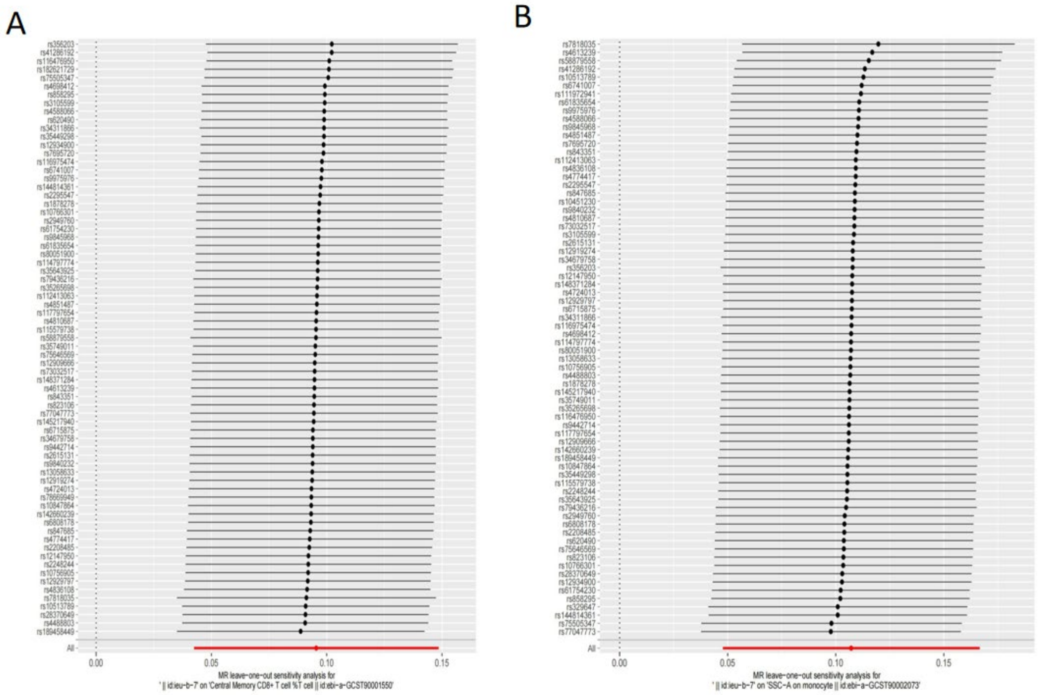

Supplement: Supplementary file 8 [file Image_1.pdf]
